# Gastric Residual Volume after Split-Dose Bowel Preparation versus Conventional Single-Dose Regimen before Anesthetic Colonoscopy

**DOI:** 10.1155/2017/6543014

**Published:** 2017-12-24

**Authors:** Shi-gui Xue, Han-lin Chen, Chun-sheng Cheng, Bao-sheng Huang, Hou-de Zhang

**Affiliations:** ^1^Department of Gastroenterology, Nanshan Hospital, Guangdong Medical University, Shenzhen 518052, China; ^2^Department of Anesthesiology, Nanshan Hospital, Guangdong Medical University, Shenzhen 518052, China

## Abstract

The aim of this study was to compare gastric residual volume (GRV) in patients given a split-dose versus a conventional single-dose of polyethylene glycol (PEG) preparation before undergoing anesthetic colonoscopy.* Methods.* In a prospective observational study, we assessed GRV in outpatients undergoing same-day anesthetic gastroscopy and colonoscopy between October 8 and December 30 of 2016. Outpatients were assigned to the split-dose (1 L PEG in the prior evening and 1 L PEG 2–4 h before endoscopy) or single-dose (ingestion of 2 L PEG ≥ 6 h before endoscopy) regimen randomly. Bowel cleansing quality was assessed with the Boston Bowel Preparation Scale (BBPS).* Results.* The median GRV in the split-dose group (17 ml, with a range of 0–50 ml; *N* = 65) was significantly lower than that in the single-dose group (22 ml, with a range of 0–62 ml; *N* = 64; *p* = 0.005), with a better bowel cleansing quality (BBPS score 8.05 ± 0.82 versus 7.64 ± 1.21; *p* = 0.028). GRV was not associated with diabetes or the use of medications.* Conclusions.* GRV after a split-dose preparation and fasting for 2–4 hours is not larger than that after a conventional single-dose preparation and fasting for 6–8 hours. The data indicates that the split-dose bowel preparation might not increase the risk of aspiration.

## 1. Introduction

Colonoscopy quality is closely related to the degree of bowel cleansing achieved. Better colon preparation results in shorter procedures, an improved cecal intubation rate, increased detection of small, large, and flat polyps, decreased patient discomfort, and reduced costs [1]. Despite the strong evidence indicating that split-dose bowel preparations are more effective than single-dose regimens, promotion of the split-dose preparation for anesthetic colonoscopy has encountered resistance. Many clinicians and anesthesiologists are concerned about possible aspiration of residual gastric fluid when a second dose is given close to the time of endoscopy. A waiting time of 6–8 hours after the last ingestion of a bowel preparation agent to start anesthesia is standard practice in many institutions. When a split-dose regimen is prescribed, patients are required to either wake up at 2:00-3:00 AM for the second dose or to undergo their procedure in the afternoon, which necessitates fasting until the afternoon for a fast of 6–8 hours to be achieved.

However, although a 6–8-hour fast is the prevailing practice to prevent the occurrence of anesthetic aspiration, this guideline does not include clear liquids. The American Society of Anesthesiologists recommends a fasting interval of only 2 hours for clear liquids before sedation [2]. Several recent studies have demonstrated that the GRV of patients who had a split-dose preparation after 2 to 3 hours was similar to or even less than that obtained after a previous-day regimen [3–5], supporting the notion that bowel preparations should be regulated as clear liquids.

The bowel preparation guide (2014) of Chinese Society of Digestive Endoscopy still calls for a preanesthetic fast of 6 hours for colonoscopy [6]. To address this issue, we conducted a prospective study comparing GRV among patients after split-dose preparation regimen versus a conventional single-dose regimen.

## 2. Material and Methods

### 2.1. Patients

This study was an open, prospective, comparison study. Between October 8 and December 30 of 2016, we recruited consecutive outpatients prospectively for elective anesthetic gastroscopy and colonoscopy on the same day at our hospital. Exclusion criteria were active gastrointestinal bleeding (i.e., melena, hematemesis, or hematochezia); suspected or known gastric outlet or bowel obstruction; a history of upper gastrointestinal surgery or colorectal surgery (excluding appendectomy or hemorrhoid surgery); severe cardiopulmonary disease; high anesthetic risk as indicated by the anesthesiologist; being pregnant or lactating; and inability to comprehend the nature of the study or give consent. This study was approved by the hospital ethics committee and all participating patients provided written informed consent.

At the time of study enrollment, patients were interviewed by a dedicated nurse who divided them into single-dose bowel preparation group or the split-dose group. The nurse carefully explained the instructions, indicating the need to be served a low-fiber diet 1 day before the procedures, and gave the patients oral and written information about forbidden foods and the importance of adequate colon cleansing for the procedure.

### 2.2. Bowel Preparation

All patients received bowel preparation with 2 L of isotonic polyethylene glycol (PEG). The single-dose bowel preparation included 2 L of PEG solution at 3:00 to 5:00 AM on the same day of the procedure, and then the patients were instructed to fast until the endoscopy was performed after at least 6 hours of fasting. The split-dose bowel preparation included 1 L of PEG at 8:00 to 9:00 PM the evening before the procedure and another 1 L of PEG between 6:00 and 7:00 AM in the morning of the procedure. Patients in the split-dose group were allowed to ingest clear liquids until the last bowel preparation intake, after which they fasted until the procedure. Fasting time was defined as the interval between the last fluid ingestion and the beginning of the procedure. Procedures could take place from 9:00 AM to 3:00 PM, with a minimum fasting time of 2 hours. Anesthetic gastroscopy was performed first, and then colonoscopy.

### 2.3. Data Collection

The patients' demographic information and basic characteristics were recorded by a dedicated nurse at the time of enrollment and before anesthetic endoscopy. Patient demographics of interest included gender, age, height, and body mass index (BMI). Basic characteristics recorded included endoscopic indications, history of diabetes, use of gastric motility inhibitors or promoters, use of proton pump inhibitors (PPIs), and the exact time of the last liquid intake.

### 2.4. Data Recorded during Endoscopic Examination: GRV, Bowel Preparation Quality, and Anesthesia-Related Adverse Events

The GRV was measured, immediately after anesthesia and insertion of the endoscope into the stomach, by suctioning all of the accumulated liquid through the suction channel of the endoscope without the addition of any water. The suctioned liquid was collected in a calibrated container and the volume of collected liquid was recorded by the endoscopy nurse. Bowel preparation quality was assessed by the colonoscopist at the end of the procedure by using the Boston Bowel Preparation Scale (BBPS). Any anesthesia-related adverse events were recorded by the anesthesiologist, including aspiration, vomiting, the need for use of a reversal agent, prolonged recovery time, or transfer to a higher-level care department.

### 2.5. Statistical Analysis

The primary outcome of interest of this study was GRV. Based on GRVs reported for single-dose preparations in the literature of 24 ± 22 ml [4] and 18.62 ± 12.73 ml [5], a sample size of 118 or 68 individuals, respectively, was estimated to be necessary to detect a 10 ml difference in mean GRV, with a power of 90% and a two-sided value of 0.05. To be prudent, we adopted the former, larger sample size. Wilcoxon rank-sum test and chi-square tests were applied to analyze the continuous variables and categorical variables, respectively. Multivariate linear regression analysis was used to assess factors that may potentially affect GRV. Univariate analysis of variables with a *p* ≤ 0.10 and variables with a clinical value were included in the analysis; *p* < 0.05 was considered to be statistically significant. Odd ratios (ORs) are reported with 95% confidence intervals (CIs). All data analyses were conducted using SPSS 19.0 (IBM, Armonk, NY).

## 3. Results

### 3.1. Patient Characteristics

In total, 129 patients were included in the study, including 65 patients who were given a split-dose bowel preparation and 64 patients accepted a single-dose regimen. All patients had a complete gastroscopy and colonoscopy. No patient had any evidence of anesthesia-related or endoscopy-related adverse events during the procedures.

The patients' demographics, clinical factors, and fasting times are reported and compared in Table 1. Briefly, there was no significant difference in mean age, sex, or body mass index between the two groups. Indications for endoscopy were also similar for the two groups. There were 15 patients in split-dose group and 16 patients in single-dose group who had no clinical symptoms and required only a physical examination. There were 21 patients and 18 patients in the groups, respectively, with constipation. The study included 3 patients with a history of diabetes; all 3 were randomly assigned to the single-dose group. In the split-dose group, 2 patients took mosapride, 2 patients took pinaverium, 1 patient took trimebutine, and 3 patients took PPIs. In the single-dose group, 1 patient took trimebutine and 6 patients took PPIs. Prevalence of diabetes and medication use did not differ significantly between the two groups. There was, as expected, a very significant difference in fasting time between the split-dose and single-dose groups.

### 3.2. Bowel Preparation Quality

The mean overall BBPS score for the split-dose group (8.05 ± 0.82) was significantly better than that of the single-dose group (7.64 ± 1.21; *p* = 0.028).

### 3.3. GRV

**Figure 1:**
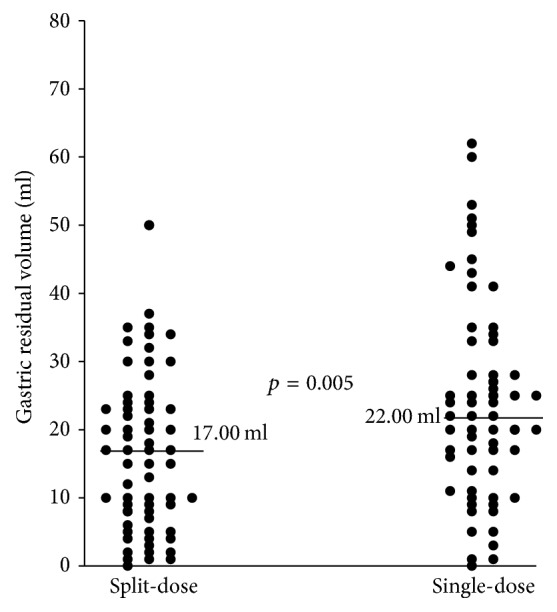
GRVs of individual patients who received the split-dose bowel preparation versus those who received the single-dose bowel preparation. The horizontal lines indicate the median GRV of each group.

As shown in Figure 1, the median GRV in patients that received the split-dose bowel preparation (17 ml with a range of 0–50 ml) was significantly less than that in patients that received the single-dose preparation (22 ml with a range of 0–62 ml; *p* = 0.005) despite the fact that the latter group fasted for 4-5 hours longer than the former group. Multivariate linear regression analysis showed that GRV was affected by fasting time (OR = 7.294; 95% CI, 0.643–3.331; *p* = 0.004), but it was not affected by the presence of diabetes, use of gastric motility inhibitors or promoters, or use of PPIs.

## 4. Discussion

In this study, we found that bowel cleansing quality was significantly better in the split-dose group than in the single-dose group. The two patient groups' characteristics were overall similar. The only significant factor in determining GRV found was fasting time. These results support advocacy for split-dose bowel preparations [1, 7].

Fears of an inadequate fasting time and higher GRV increasing the risk of aspiration account for the main resistance to prescribing split-dose bowel preparations. Thus, it is particularly noteworthy that, even with their markedly shorter fasts before the endoscopy (minimum, 2 hours), the patients in the split-dose preparation group had a lower, rather than a higher, GRV than the single-dose group. These results are similar to those of studies conducted in the USA and Spain showing that the GRV of patients given a split-dose preparation was less than or not different from the GRV of patients given a single-dose bowel preparation [3–5]. Our findings also fit those of a prior report showing a smaller intraoperative gastric volume in patients with a shortened versus a standard-length fast before elective surgeries [8].

Given that no patient had any evidence of anesthesia-related or endoscopy-related adverse events during the procedures, this study supports the notion that PEG solution can be treated as a clear liquid and thus be considered safe up to 2 hours before anesthetic endoscopy [2]. A reduced fasting time of only 2 hours is more acceptable and less burdensome to patients than a 6-hour fast. Moreover, in addition to reducing the fasting time requirement, the split-dose preparation regimen does not require patients to get up in the middle of the night or endure being hungry throughout most of the day into the afternoon. In addition, the split-dose preparation regimen reduces the amount of liquid that must be consumed at once.

Gastric emptying is controlled by the enteric nervous system in response to physical and chemical stimulation signaling the presence of food as well as modulatory influences from the autonomic nervous system, the central nervous system, and endocrine signaling [9]. Therefore, GRV is dependent on factors besides fasting time, including factors involved in gastric emptying. For example, in a certain range, gastric emptying is proportional to the volume of stomach contents, such that the more one has eaten, the faster his or her stomach empties. The specific mechanism underlying the smaller GRV in the split-dose group, relative to the single-dose group, is unknown; however, it can be presumed that it is consequent to complex interactive regulation of gastric emptying and gastric juice secretion.

Although we attended to the question of whether factors with the potential to suppress gastric emptying and gastric juice secretion (e.g., diabetes, medications) affected GRV, our multiple regression analysis did not find any significant influence of these potential influences. Although these factors were only relevant for small portions of our groups, similar studies with higher proportions of patients affected by these factors have reported similar negative results [3–5]. Thus, together, these findings suggest that patients with gastric motility disorders may not need special extended fasting times before anesthesia. It should be noted that although GRV is related to the occurrence of anesthesia aspiration, there are a number of other important factors including the patient's clinical characteristics, anesthesia depth, and anesthesia monitoring [10]. Therefore, regardless of GRV, strict aspiration prevention and control measures are essential.

Although we found that it is safe to administer anesthesia 2 hours after ingestion of a bowel preparation, this finding does not mean that 2 hours is the best, most clinically efficient interval. It may be preferable to require a longer fasting period for outpatients to avoid the need to use the restroom on the way to the hospital. Moreover, bowel preparation quality has been reported to be optimal with a 4–6-hour interval from ingestion of the last dose of preparation solution to the procedure [11, 12]. Hence, clinicians may suggest a particular fast period in the period of 2 hours to 6 hours depending on the particular patient's circumstances, such as whether the patient is hospitalized, an outpatient, or in emergency care and other practical considerations.

This study had some limitations, including nonenrollment of inpatients and patients with severe gastric motility disorders and a lack of double-blinding. However, the cohort was representative of the majority patients who undergo this procedure in terms of their clinical condition.

In conclusion, GRV in patients subjected to anesthetic endoscopy after a split-dose preparation and fasting for 2 hours was found to be significantly lower than that after a conventional single-dose regimen and fasting for 6 hours. We can conclude that PEG solution behaves as a standard clear liquid, and thus physicians need not wait longer than 2 hours after the last ingestion of bowel preparation to proceed with anesthetic endoscopy. Additionally, the split-dose preparation regimen yields very good cleansing quality while reducing the volume of rapidly ingested liquid, disruptions to patients' sleep, and the duration of patient hunger. Hence, the split-dose regimen improves the comfort of preparation for patients, which in itself is a worthwhile consideration. Given these observations, we agree with Pochapi's assertion that “it's time to take the split-standard out of the split-prep” [13].

## Figures and Tables

**Table 1:** Patient demographics, medications, and fasting time.

Variable	Split-dose group *N* = 65	Single-dose group *N* = 64	*p*
Mean age, years	42.8 ± 8.9	41.9 ± 9.6	0.577
Sex, number of males/number of females	31/34	36/28	0.331
Body mass index	22.8 ± 3.5	23.4 ± 2.8	0.321
*Indications for procedures, N*			
Asymptomatic	15	16	0.798
Diabetes	0	3	0.237
Constipation	21	18	0.605
*Medication use, N*			
Antispasmodic	2	0	0.496
Gastroprokinetic agent	2	1	>0.999
PPIs	3	6	0.474
Fasting time, hours	2.7 ± 0.6	6.2 ± 0.2	<0.001
